# The Highly Conserved Asp23 Family Protein YqhY Plays a Role in Lipid Biosynthesis in *Bacillus subtilis*

**DOI:** 10.3389/fmicb.2017.00883

**Published:** 2017-05-19

**Authors:** Dominik Tödter, Katrin Gunka, Jörg Stülke

**Affiliations:** Department of General Microbiology, Institute of Microbiology and Genetics, University of GöttingenGöttingen, Germany

**Keywords:** fatty acid biosynthesis, Asp23 family, DUF322, protein localization, ACCase

## Abstract

In most bacteria, fatty acid biosynthesis is an essential process that must be controlled by the availability of precursors and by the needs of cell division. So far, no mechanisms controlling synthesis of malonyl-coenzyme A (CoA), the committed step in fatty acid synthesis, have been identified in the Gram-positive model bacterium *Bacillus subtilis*. We have studied the localization and function of two highly expressed proteins of unknown function, YqhY and YloU. Both proteins are members of the conserved and widespread Asp23 family. While the deletion of *yloU* had no effect, loss of the *yqhY* gene induced the rapid acquisition of suppressor mutations. The vast majority of these mutations affect subunits of the acetyl-CoA carboxylase (ACCase) complex, the enzyme that catalyzes the formation of malonyl-CoA. Moreover, lack of *yqhY* is accompanied by the formation of lipophilic clusters in the polar regions of the cells indicating an increased activity of ACCase. Our results suggest that YqhY controls the activity of ACCase and that this control results in inhibition of ACCase activity. Hyperactivity of the enzyme complex in the absence of YqhY does then provoke mutations that cause reduced ACCase activity.

## Introduction

The Gram-positive model bacterium *Bacillus subtilis* belongs to the most-studied and best understood organisms. However, of its about 4,100 genes 800 encode proteins of unknown function (Michna et al., 2016)^1^. Thus, there is still a requirement for future research to fully understand the biology of *B. subtilis*. An important step in the analysis of any organism is the identification of the set of essential genes as well as of those genes that are required for a minimal genome. In *B. subtilis*, recent studies suggest that 251 protein-coding genes are essential, i.e., they cannot be deleted individually (Kobayashi et al., 2003; Commichau et al., 2013; Reuß et al., 2016). In *B. subtilis*, many proteins have paralogs which may be redundant or have specialized functions. Specifically, 351 gene pairs were detected in the *B. subtilis* genome. For several of the corresponding gene pairs it has been shown that the individual genes are dispensable whereas the common function exerted by both proteins is essential (Thomaides et al., 2007).

A recent re-evaluation of the essential gene set of *B. subtilis* revealed that there are left only six essential proteins of unknown function (Commichau et al., 2013). Among these genes are *yloU* and *yqhY* which encode the paralogous proteins YloU and YqhY. Both proteins contain the conserved domain of unknown function DUF322 and are members of the so-called Asp23 family. In an analysis of the *B. subtilis* gene pairs, both genes were reported to be essential (Thomaides et al., 2007). The members of the Asp23 family are ubiquitously present in Gram-positive bacteria, i.e., the Firmicutes and Actinobacteria, but they are also found in the *Deinococcus*/*Thermus* group as well as in the *Chlamydia*, *Thermotoga*, and *Bacteroides*/*Fusobacterium* phyla. Interestingly, these proteins are always encoded with at least two copies in each genome. The name-giving representative of the family is the *Staphylococcus aureus* Asp23 protein, which was found to be induced after alkaline shock (Kuroda et al., 1995). With about 25,000 molecules per cell, this protein belongs to the most abundant proteins in *S. aureus* even during logarithmic growth (Maass et al., 2011). Recently, it has been reported that Asp23 is a membrane-bound protein and that deletion of the gene provokes a cell envelope stress response (Müller et al., 2014). The *B. subtilis yloU* and *yqhY* genes are also strongly constitutively expressed (Nicolas et al., 2012). The high expression of *yloU* and *yqhY* is rather unusual for genes of unknown function. A recent analysis of the *B. subtilis* revealed that the 20% of proteins of unknown function encoded in the genome account for only 4% of the cellular translation activity (Reuß et al., 2017). The strong conservation of the *yloU*/*yqhY* gene pair, the essentiality of the genes as well as their exceptionally high expression suggest that the encoded proteins play important roles in the physiology of *B. subtilis* and other Gram-positive bacteria.

**FIGURE 1:**
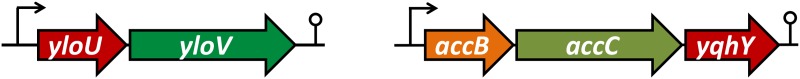
**Genetic context of *yqhY* and *yloU*.** The genes are located in two distinct operons together with genes encoding proteins involved in fatty acid metabolism. *yqhY* clusters with *accB* (biotin carboxyl carrier protein) and *accC* (biotin carboxylase subunit). *yloU* forms an operon with *yloV*.

In *B. subtilis* as well as in many other Firmicutes, the *yloU* gene is part of a conserved operon with *yloV* (see **Figure 1**), which encodes another protein of unknown function. The *yqhY* gene is the distal gene of the tricistronic *accBC-yqhY* operon (Nicolas et al., 2012; see **Figure 1**). The *accB* and *accC* genes encode the AccB and AccC subunits of the acetyl-coenzyme A (CoA) carboxylase (ACCase) complex. ACCase catalyzes the formation of malonyl-CoA, the first committed step in fatty acid biosynthesis. The *accBC-yqhY* operon organization is conserved not only in the Firmicutes, but also in *Chlamydia*, *Deinococcus*/*Thermus*, and Fusobacteria, but not in the Actinobacteria. As conserved gene clustering is often an indication of related protein functions, and even physical interactions between the encoded proteins (Dandekar et al., 1998; Meyer et al., 2011), it is tempting to speculate that YqhY might play some role in the control of fatty acid biosynthesis.

Fatty acid biosynthesis is an essential process in nearly all bacteria. As mentioned above, the first step is the carboxylation of acetyl-CoA to malonyl-CoA. In the next step, the CoA is replaced by acyl carrier protein (ACP). Malonyl-ACP serves as the universal substrate for fatty acid elongation by the consecutive addition of acetyl-moieties, releasing CO_2_. The ACCase complex is composed of four subunits that are organized in two sub-complexes, AccB–AccC and AccA–AccD. The first step of the reaction is the carboxylation of biotin by the biotin carboxylase (AccC). The biotin cofactor is attached to the biotin carboxyl carrier protein (AccB; Marini et al., 1995). Finally, the carboxyl group is transferred to acetyl-CoA by the carboxyltransferase domain (AccAD) of the ACCase (Cronan and Waldrop, 2002). Due to their key role in membrane biosynthesis, all subunits of ACCase are essential, and have been proposed to be attractive drug target candidates (Tong, 2005).

Here, we have re-evaluated the essentiality of the *yloU* and *yqhY* genes in *B. subtilis*. While both genes were found to be dispensable, the *yqhY* mutant readily acquired secondary suppressor mutations. Whole genome sequencing indicated that such mutations affect any of the subunits of ACCase supporting the idea of a functional link between YqhY and fatty acid biosynthesis. Moreover, the accumulation of lipophilic clusters in the *yqhY* mutant confirms a role for YqhY in the control of lipid metabolism.

## Materials and Methods

### Bacterial Strains and Growth Conditions

The *B. subtilis* strains used in this study are listed in **Table 1**. *Escherichia coli* DH5α (Sambrook et al., 1989) was used for plasmid constructions and transformation using standard techniques (Sambrook et al., 1989). Luria–Bertani (LB) broth was used to grow *E. coli* and *B. subtilis*. When required, media were supplemented with antibiotics at the following concentrations: ampicillin 100 μg/ml (for *E. coli*) and kanamycin (10 μg/ml), chloramphenicol (5 μg/ml), or spectinomycin 150 μg/ml (for *B. subtilis*). SP (sporulation) plates were prepared by the addition of 15 g/l Bacto agar (Difco) to SP medium (8 g of nutrient broth per liter, 1 mM MgSO_4_, 13 mM KCl, supplemented after sterilization with 2.5 μM FeSO_4_, 500 μM CaCl_2_, and 10 μM MnCl_2_). *B. subtilis* was transformed with plasmid DNA according to the two-step protocol (Kunst and Rapoport, 1995). Transformants were selected on SP plates containing antibiotics as above.

**Table 1:** *Bacillus subtilis* strains used in this study.

Strain	Genotype	Source^a^
168	*trpC2*	Laboratory collection
GP1465	*trpC2 ΔyloU::cat*	This work
GP1467	*trpC2 ΔyloUV::cat*	This work
GP1468	*trpC2 ΔyqhY::ermC*	This work
GP1469	*trpC2 ΔyqhY::ermC accD*_G283V_	Suppressor mutant, isolated from GP1468
GP1470	*trpC2 ΔyloU::cat ΔyqhY::ermC accA*_H105P_	This work
GP1471	*trpC2 yqhY-gfp spc*	pGP1482 → 168
GP1472	*trpC2 yloU-gfp spc*	pGP1483 → 168
GP1487	*trpC2 accA-3xFLAG spc*	pGP1499 → 168
GP1488	*trpC2 ΔyqhY::ermC accD*_G283V_ *accA-3xFLAG spc*	pGP1499 → GP1469
GP1489	*trpC2 ΔyloU::cat ΔyqhY::ermC accA*_H105P_ *accA-3xFLAG spc*	pGP1499 → GP1470
GP1765	*trpC2 ΔyqhY::cat*	This work
GP2321	*trpC2 ΔyqhY::ermC accD*_L176F_	Suppressor mutant, isolated from GP1468
GP2322	*trpC2 ΔyqhY::ermC accD*_A229S_	Suppressor mutant, isolated from GP1468
GP2323	*trpC2 ΔyqhY::ermC accD*_I38N_	Suppressor mutant, isolated from GP1468
GP2601	*trpC2 ΔyqhY::ermC accB*_L2S_	Suppressor mutant, isolated from GP1468
GP2602	*trpC2 ΔyqhY::ermC accC*_E12A_	Suppressor mutant, isolated from GP1468
GP2603	*trpC2 ΔyqhY::ermC accC*_V 98A_	Suppressor mutant, isolated from GP1468
GP2604	*trpC2 ΔyqhY::ermC accC*_F192L_	Suppressor mutant, isolated from GP1468


^a^*Arrows indicate construction by transformation*.

### Plasmid Constructions

All plasmids used in this study are listed in **Table 2**. To express *B. subtilis* proteins under the control of their native expression signals, fused to the monomeric green fluorescent protein (GFP), we used plasmid pBP43 which allows the construction of GFP fusions and integration of the plasmid into the chromosome via Campbell-type recombination between the cloned gene fragment and the chromosomal copy of the gene (Cascante-Estepa et al., 2016). For the fusion of YloU and YqhY to the GFP, we amplified the 3′ 600 bp (lacking a stop codon) of each gene, with the respective oligonucleotide pair (see Supplementary Table S1), using chromosomal DNA of *B. subtilis* 168 as the template. The PCR products were cloned between the *Bam*HI and *Pst*I sites of pBP43. The resulting plasmids were pGP1482 and pGP1483 for *yqhY* and *yloU*, respectively. Integration of these plasmids into the chromosome of *B. subtilis* leads to the in-frame fusion of the *gfp* alleles to the entire genes lacking their stop codon.

**Table 2:** Plasmids used in this study.

Plasmid	Relevant characteristics	Primers	Reference
pBP43	Construction of gfp fusions		Cascante-Estepa et al., 2016
pGP1331	Addition of FLAG tags		Lehnik-Habrink et al., 2010
pGP1482	pBP43-*yqhY*	DT31/DT32	This study
pGP1483	pBP43-*yloU*	DT23/DT30	This study
pGP1499	pGP1331-*accA*	DT64/DT66	This study


To allow immunological detection of AccA, a triple FLAG tag was fused to the protein. For this purpose, we used the vector pGP1331 (Lehnik-Habrink et al., 2010). Briefly, a 600 bp 3′ fragment of the *accA* gene was amplified by PCR and cloned into pGP1331. The resulting plasmid was pGP1499. This plasmid was then used to transform the relevant *B. subtilis* strains in order to introduce the fusion into the chromosome. The designations of the resulting strains are listed in **Table 1**.

### Construction of Deletion Strains

Deletion of the *yloU* and *yqhY* genes was achieved by transformation with PCR products constructed using oligonucleotides (see Supplementary Table S1) to amplify DNA fragments flanking the target genes and intervening antibiotic resistance cassettes as described previously (Guérout-Fleury et al., 1995; Wach, 1996).

### Microscopy

For fluorescence microscopy, cells were grown at 37°C in LB medium to an OD_600_ of 0.5–1.0 (unless otherwise indicated), harvested by centrifugation and resuspended in phosphate-buffered saline (50 mM; pH 7.5). The membrane was stained with Nile Red (Sigma, Darmstadt, Germany) (10 μg/ml). The cells were spotted onto a microscope slide covered with a film of 1% agarose in water. Fluorescence images were obtained with an AxioImager M2 fluorescence microscope, equipped with digital camera AxioCam MRm and AxioVision Rel 4.8 software for image processing and an EC Plan-NEOFLUAR 100×/1.3 objective (Carl Zeiss, Göttingen, Germany). The filter set 37 (BP 450/50, FT 480, BP 510/50; Carl Zeiss) and the set 43 (BP 545/25, FT 570, BP 605/70; Carl Zeiss) were applied for GFP and Nile Red detection, respectively. Images were taken with an exposure time of 1 s for the GFP constructs and 500 ms for visualization of the Nile Red stain. Pictures of *B. subtilis* colonies on agar plates were taken with a stereo microscope Lumar.V12 (Carl Zeiss) equipped with the ZEN lite 2011 (blue edition) software.

### Genome Sequencing

Chromosomal DNA from *B. subtilis* was isolated using the peqGOLD Bacterial DNA Kit (Peqlab, Erlangen, Germany). To identify the mutations in the suppressor mutant strains GP1469 and GP1470 (see **Table 1**), the genomic DNA was subjected to whole-genome sequencing. The reads were mapped on the reference genome of *B. subtilis* 168 (GenBank accession number: NC_000964) (Barbe et al., 2009). Mapping of the reads was performed using the Geneious software package (Biomatters Ltd., New Zealand; Kearse et al., 2012). Single-nucleotide polymorphisms were considered as significant when the total coverage depth exceeded 25 reads with a variant frequency of ≥90%. All identified mutations were verified by PCR amplification and Sanger sequencing.

### Determination of AccA Protein Expression by Western Blot Analysis

To monitor the amounts of the AccA-FLAG protein, the strains were grown in LB medium and harvested in the logarithmic phase of growth (OD_600_ of 1.0). The cells were disrupted using a French press and 6 μg crude extract of each culture were loaded on a 12% sodium dodecyl sulfate-polyacrylamide gel. Total protein concentration was determined with the Bradford method (Roti^®^-Quant, Carl Roth, Karlsruhe, Germany). Following electrophoresis, the proteins were transferred onto a polyvinylidene difluoride membrane (Bio-Rad) by electroblotting. Rabbit anti-FLAG polyclonal antibodies served as primary antibodies. They were visualized by using anti-rabbit immunoglobulin alkaline phosphatase secondary antibodies (Promega) and the CDP-Star detection system (Roche Diagnostics), as described previously (Schmalisch et al., 2002).

## Results

### Deletion of the *yloU* and *yqhY* Genes

In a previous study, the two Asp23 family proteins of *B. subtilis* were reported to be essential (Thomaides et al., 2007). In an attempt to unravel the functions of the unknown essential genes, we decided to verify this result, and thus to attempt deletion of the *yloU* and *yqhY* genes. Surprisingly, deletion of both genes was possible indicating that they are dispensable. Thus, our result was in agreement with the original study on gene essentiality in *B. subtilis* in which both genes have been inactivated. Moreover, we sought to determine whether a double mutant lacking both Asp23 family proteins would also be viable. Indeed, the double mutant strain GP1470 could be constructed on rich medium. However, we observed the appearance of suppressor mutations. Similarly, the *yqhY* mutant GP1468 gave rise the appearance of suppressor mutants. In contrast, the *yloU* as well as the *yloUV* mutant strains, GP1465 and GP1467, respectively, grew on SP plates indistinguishable from the isogenic wild type strain *B. subtilis* 168. These results indicate that both genes that encode proteins of the Asp23 family, are dispensable in *B. subtilis*. However, YqhY seems to be important for the physiology of the cells as indicated by the rapid lysis and the appearance of suppressor mutations.

### Localization of YloU and YqhY

**FIGURE 2:**
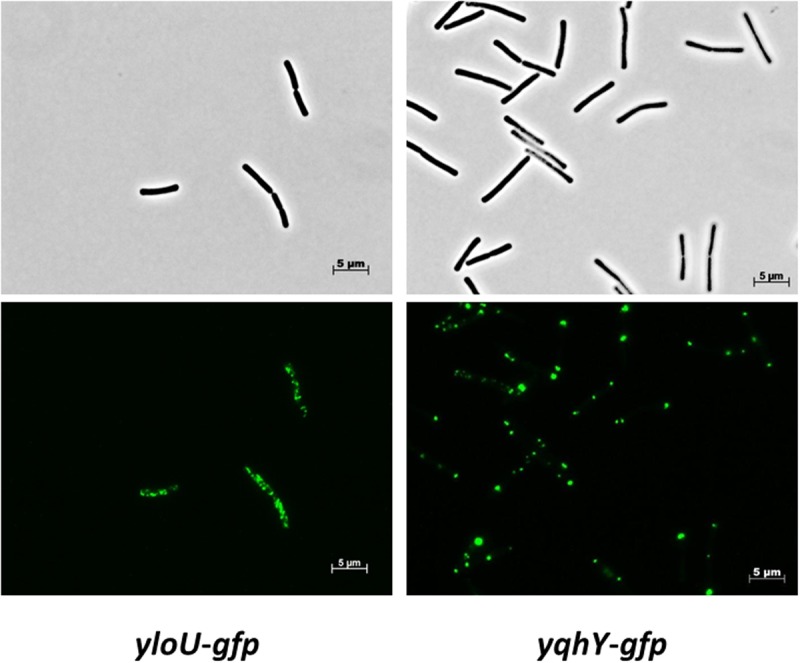
**Localization of YloU and YqhY.** The proteins were fused to a monomeric GFP at the C-terminus and visualized via fluorescence microscopy.

To get more insights into the physiological roles of the two proteins, we determined their subcellular localization. For this purpose, we fused the proteins to the monomeric GFP, and expressed the fusion proteins under the control of the native promoters to avoid artificial overexpression resulting in aggregation and mislocalization. Strain GP1471 encoding YqhY-GFP as the only copy of YqhY was stable, thus indicating that the fusion protein was biologically active. For localization studies, the strains were grown in SP medium and exponentially growing cells were observed by fluorescence microscopy (see **Figure 2**). For YloU, we found the formation of spots throughout the cytoplasm. In contrast, YqhY localized to the cell poles. Our localization studies indicate that YloU and YqhY are unlikely to physically and functionally interact in logarithmically growing cells since they localize to distinct areas in the cell.

### Isolation and Analysis of Suppressor Mutations in the *yqhY* Mutant Background

**FIGURE 3:**
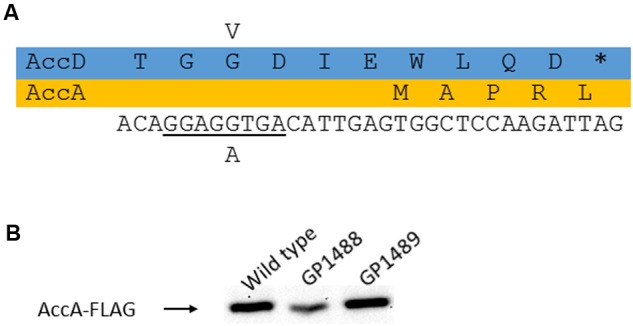
**Analysis of the AccD-G283V substitution. (A)** Effect of the mutation on the *accD* coding region and the AccD protein as well as on the *accA* ribosome binding site. **(B)** Expression of AccA as a consequence of the altered ribosome binding site. To study AccA levels, the protein was fused to a FLAG tag.

As mentioned above, we observed the appearance of suppressor mutations when the *yqhY* gene had been deleted. For two of these suppressor strains that had been isolated from the *yqhY* single mutant (GP1469) and from the *yloU yqhY* double mutant (GP1470), the mutations were identified by whole genome sequencing. Both mutant strains carried single mutations in the *accDA* operon thus affecting the AccA and AccD subunits of the ACCase complex. Specifically, we found substitutions in AccD (G283V) and AccA (H105P) in GP1469 and GP1470, respectively. Interestingly, the former mutation not only results in an amino acid substitution in AccD, but also affects the ribosomal binding site for the *accA* open reading frame suggesting reduced expression of the AccA subunit (see **Figure 3A**). To test whether this mutation in GP1469 does indeed affect the cellular amounts of AccA, we constructed an isogenic set of strains in which a FLAG tag was attached to the AccA protein to facilitate immunological detection. As shown in **Figure 3B**, the amounts of AccA were reduced in the strain carrying the *accD*_G283V_ allele. In contrast, no reduction of AccA levels was observed in the strain with the *accA*_H105P_ allele. These results demonstrate that the mutation of the *accA* ribosomal binding site results in reduced expression of the protein.

As the ACCase complex (including each individual subunit) is essential for *B. subtilis*, these mutations are unlikely to inactivate the enzyme. In order to get further insights into the link between YqhY and ACCase, we isolated another set of 10 suppressor mutants, based on the *yqhY* mutant, GP1468. For these mutants, the *accDA* and *accBC* operons encoding the subunits of the ACCase complex were amplified by PCR and sequenced. In eight of the mutant strains, we found mutations in one of the four genes. Interestingly, each suppressor mutant harbored only one single amino acid substitution in one of the ACCase subunits. The mutations were scattered over the ACCase subunits, and they affected both highly conserved and less conserved regions. Interestingly, we did not obtain a single mutant strain that encoded truncated versions of an ACCase subunit. Truncations are typically encountered if the suppressor mutations are due to gene inactivation (Zaprasis et al., 2014; Gundlach et al., 2015b; Rosenberg et al., 2016). The absence of truncations in the ACCase subunits is in excellent agreement with the essential function of this complex for fatty acid biosynthesis. Thus, we can conclude that the suppressor mutations affect the activity of ACCase, but do not result in inactivation of the enzyme.

**FIGURE 4:**
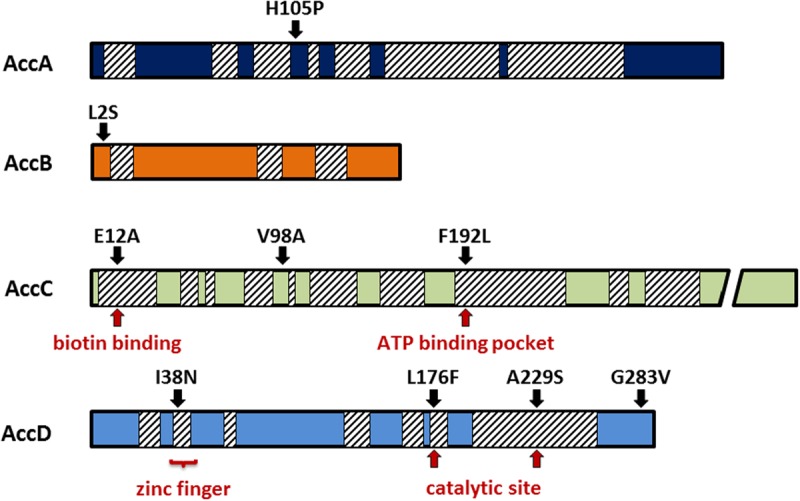
**Mutations in the acetyl-CoA carboxylase subunits.** All mutations were single substitutions obtained from distinct clones. Many of them were located in highly conserved regions (hatched boxes) responsible for substrate binding or catalytic activity.

The distribution of the suppressor mutations is shown in **Figure 4**. We have isolated one mutation in AccA (H105P, see above), one mutation at the very N-terminus in the biotin carrier protein AccB (L2S), three mutations in AccC (E12A, V98A, F192L). Intriguingly, the residues E12 and F192 are highly conserved from archaeal and bacterial to the eukaryotic enzymes. The residue E12 is located in close proximity to the biotin binding site of AccC (Chou et al., 2009), whereas F192 is close to the ATP-binding pocket (Waldrop et al., 2012). Four distinct mutations were found in AccD (I38N, L176F, A229S, G283V). I38 is part of the conserved zinc finger motif. However, while the isoleucine is not strictly conserved at this position, the asparagine may alter the protein’s structure and activity. In contrast, L176 and A229 are highly conserved residues, and L176 is part of the active center of the enzyme (Bilder et al., 2006). Taken together, single mutations all over the ACCase subunits suppress the growth defect of the *yqhY* mutation.

### Formation of Lipophilic Clusters in the Absence of YqhY

**FIGURE 5:**
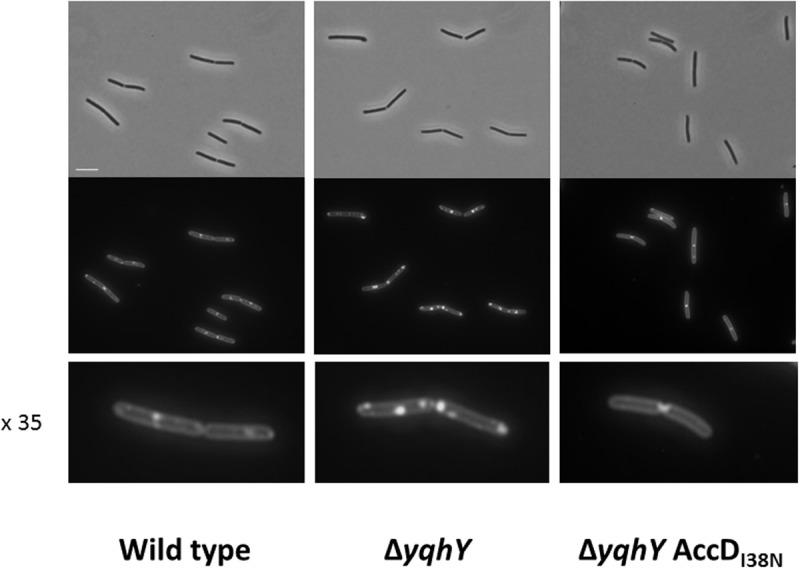
**Staining of lipophilic regions.** Samples were taken at exponential growth phase and treated with the membrane dye Nile Red. The lowest panel shows individual cell in 35-fold magnification. Scale bar, 5 μm.

The results presented above suggest that YqhY may interfere with fatty acid, and thus ultimately with membrane lipid biosynthesis. To test this idea, we stained the membranes of *B. subtilis* with the fluorescent dye Nile Red (Strahl et al., 2014). For the wild type strain, staining of the membrane was observed. In contrast, lipophilic clusters at the cell poles were found in the *yqhY* mutant strain GP1765 (see **Figure 5**). We also tested the membranes of one of the suppressor mutants, GP2323 (AccD_I38N_). In this strain, the formation of the lipophilic clusters was abolished. Thus, the membrane stain strongly supports the idea that YqhY has an impact on lipid biosynthesis in *B. subtilis*. In the absence of YqhY, fatty acid biosynthesis seems to be intensified.

### The Hyperactive Acetyl-CoA Carboxylase Drains the Cellular Acetyl-CoA Pool

**FIGURE 6:**
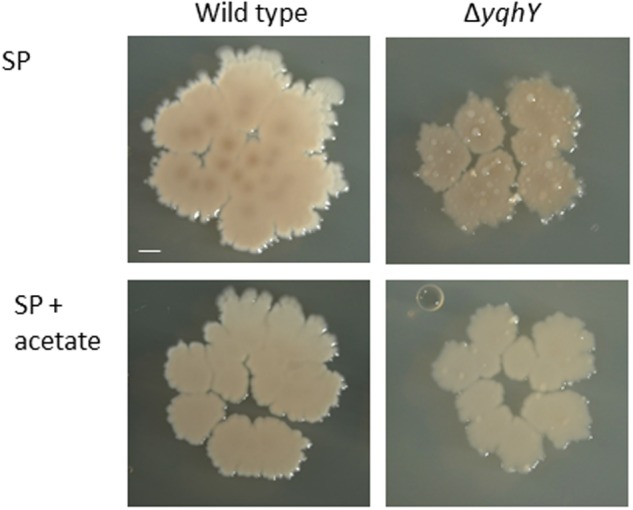
**Effect of acetate on the yqhY mutant.** The wild type strain 168 and the isogenic yqhY mutant GP1468 were cultivated on SP plates without or with added acetate (0.5%). Scale bar, 1 mm.

As shown above, ACCase activity seems to be increased in the absence of YqhY. This might result in depletion of the cellular acetyl-CoA pool. If this were the case, one would expect that addition of acetate to the medium would that the *yqhY* mutant would be more stable if provided with external acetate. To test this hypothesis, we plated the wild type strain 168 and the isogenic *yqhY* mutant GP1468 on SP plates with and without added acetate (see **Figure 6**). While the acetate had no effect on growth of the wild type strain, the acquisition of suppressor mutations was substantially reduced in the *yqhY* mutant, indicating that acetate or more likely acetyl-CoA becomes limiting in the absence of YqhY.

## Discussion

In this work, we provide the first detailed analysis of Asp23 family proteins in the Gram-positive model bacterium *B. subtilis*. Members of this protein family are ubiquitous in many bacterial phyla, and they are highly expressed in different bacteria such as *B. subtilis* and *S. aureus*. These features suggest that proteins of this family are very important for the organisms that encode them. Despite this apparent relevance, only one previous study has addressed the properties of an Asp23 family member, i.e., the name-giving Asp23 protein from *S. aureus* (Müller et al., 2014). Here, we have unraveled an intimate link between the highly conserved Asp23 family protein YqhY and membrane lipid biosynthesis in *B. subtilis*.

Fatty acid biosynthesis is essential for most bacteria, with the notable exception of few pathogenic bacteria that acquire their fatty acids from the host cells. The first step in fatty acid biosynthesis, the formation of malonyl-CoA, is catalyzed by the ACCase multiprotein complex (Cronan and Waldrop, 2002). Lipid metabolism must be closely coordinated with cell division to provide the amounts of fatty acids that precisely meet the requirements of growing or non-growing cells. Previous studies with *B. subtilis* have identified the FadR and FapR regulator proteins that control the expression of fatty acid degradative and biosynthetic genes, respectively (Fujita et al., 2007). Both regulators respond to metabolic signals, i.e., the accumulation of long chain acyl-CoA and malonyl-CoA, respectively (Martinez et al., 2010; Fujihashi et al., 2014). The regulation by late or early intermediates in fatty acid biosynthesis indicates that FadR and FapR are responsible to match the expression of fatty acid metabolic genes to the availability and requirements of fatty acids, but that these regulators may not be involved in linking fatty acid metabolism to cell division. Moreover, while FapR controls the expression of fatty acid biosynthesis genes and operons, the *accDA* and *accBC yqhY* operons are expressed constitutively and are not members of the FapR regulon (Schujman et al., 2003). These findings suggest a different control of malonyl-CoA biosynthesis.

For most metabolic pathways, the initial steps are major targets for regulation, often at the level of transcription. This statement is in obvious contradiction to the lack of described regulatory mechanisms for the genes encoding the ACCase complex in *B. subtilis*. However, recent work has identified control of ACCase enzyme activity by a regulatory interaction of the biotin carboxyl carrier protein (AccB) with a so-called PII protein in plants, Cyanobacteria, and Proteobacteria (Feria Bourellier et al., 2010; Rodrigues et al., 2014; Gerhardt et al., 2015; Hauf et al., 2016). The PII proteins are small signal transduction proteins that control ammonia uptake and provide a regulatory link between carbon and nitrogen metabolism (Forchhammer and Lüddecke, 2016). This regulatory interaction results in an inhibition of ACCase activity specifically under conditions of low carbon source availability (Gerhardt et al., 2015). Interestingly, of the two PII proteins of *E. coli*, only GlnB, but not GlnK interacts with AccB. *B. subtilis* possesses one PII protein, NrgB, and a PII-like protein, DarA. While NrgB is the ortholog of GlnK, DarA is a cyclic di-AMP-binding protein of unknown function (Detsch and Stülke, 2003; Gundlach et al., 2015a). There is no indication for a role of these proteins in the control of ACCase activity in *B. subtilis*. Thus, it seems reasonable that other factors control ACCase expression and/or activity.

The results presented in this study suggest that YqhY may be such a novel regulator of ACCase activity in *B. subtilis* and in other bacteria that do not possess a PII to interact with AccB. Indeed, inactivation of *yqhY* specifically provoked a variety of mutations in all subunits of ACCase. This observation indicates the functional relation between YqhY and ACCase which is also supported by the conserved *accBC yqhY* operon structure (see **Figure 1**). How might YqhY affect the activity of ACCase? If the mutations would result in enhanced ACCase activity, they would not be likely to affect conserved regions of the enzyme. However, this is what we have observed. Thus, the mutations probably result in reduced ACCase activity. The essentiality of the enzyme complex as well as well as the lack of frame shift mutations that would result in inactive truncated proteins are in excellent agreement with the idea that the mutations reduce but do not abolish ACCase activity. Thus, the *yqhY* mutant seems to suffer from ACCase hyperactivity. This conclusion is further substantiated by the observation that the *yqhY* mutant accumulates lipids at the cell poles, and that this lipid accumulation does not take place in a suppressor mutant with a substitution in AccD. Intriguingly, enhanced lipid body production was also observed in a *Chlamydomonas reinhardtii* strains with reduced level of the PII protein and the resulting increased ACCase activity (Zalutskaya et al., 2015). Thus, it is tempting to speculate that YqhY takes over one of the multiple PII functions, i.e., the control of ACCase activity.

As mentioned above, lipid biosynthesis must be coupled to the requirements of cell division. YqhY is a candidate for linking cell division and ACCase activity: The YqhY protein is recruited to the polar regions of the cell (see **Figure 2**). Further research will address the molecular mechanisms that link YqhY and ACCase activity. Three hypotheses will be followed: (i) YqhY might directly control the enzymatic activity of ACCase, (ii) YqhY might control the localization of ACCase, or (iii) YqhY might affect ACCase stability. The former hypothesis is supported by the high expression of YqhY. In the latter two cases, the effect of YqhY on ACCase activity would be rather indirect. A control of ACCase localization is supported by the polar localization of YqhY. In the absence of this protein, the ACCase complex might re-localize to the cell poles as indicated by the formation of polar lipid clusters in the *yqhY* mutant strain. However, the approval of one of the above ideas will be the subject of our future work.

## Author Contributions

DT and JS designed the study. DT and KG performed the experiments. JS, KG, and DT wrote the paper.

## Conflict of Interest Statement

The authors declare that the research was conducted in the absence of any commercial or financial relationships that could be construed as a potential conflict of interest.
