# Supplementary material for: The Highly Conserved Asp23 Family Protein YqhY Plays a Role in Lipid Biosynthesis in Bacillus subtilis
Source: Front Microbiol. 2017 May 19;8:883. doi: 10.3389/fmicb.2017.00883 (PMC5437119; doi:10.3389/fmicb.2017.00883)
Supplement: Supplementary file 1 [file Table_1.DOCX]

**Table S1:** Primers used in this study.

| **Primer** | **Sequence**  restriction sites are underlined | **Restriction**  **sites** |
| --- | --- | --- |
| **Construction of *yqhY*, *yloU* and *yloUV* mutants** | |  |
| ***yqhY*-mutant** | |  |
| DT02 | 5‘‑CCGGGTTCACAGGGAATTATAGAAAATG |  |
| DT03 | 5‘‑CCTATCACCTCAAATGGTTCGCTGCTTACCCAAATGCGTATCTTCGTGATC |  |
| DT04 | 5‘‑CGAGCGCCTACGAGGAATTTGTATCGCACATCGTCGGCATTCAATTTGACAC |  |
| DT05 | 5‘‑TACATCTTTATCAGGGGAGATACGTTCG |  |
| ***yloU*-mutant** | |  |
| DT08 | 5‘‑TTCATGTGTATCAAGCAGAAAAAGATCAAACAG |  |
| DT09 | 5‘‑CCTATCACCTCAAATGGTTCGCTGCGCGATGACTTCATTAGATATATCAATCTG |  |
| DT10 | 5‘‑CGAGCGCCTACGAGGAATTTGTATCGGGCAGTGGATTCTGTCAATATTTATGTCC |  |
| DT11 | 5‘‑GCTGTTTTGCCGGCGGTGTTTC |  |
| ***yloUV*-mutant** | |  |
| DT08 | 5‘‑TTCATGTGTATCAAGCAGAAAAAGATCAAACAG |  |
| DT09 | 5‘‑CCTATCACCTCAAATGGTTCGCTGCGCGATGACTTCATTAGATATATCAATCTG |  |
| DT16 | 5‘‑CGAGCGCCTACGAGGAATTTGTATCGGATCCACAATGGGAAACAGCCTCTG |  |
| DT17 | 5‘‑GCCTGTTAAGCCCGTTTGGCTC |  |
| **triple FLAG constructions** | |  |
| ***accA*-3xFLAG** | |  |
| DT64 | 5‘‑AAAGGATCCCCTAGTCCGCAATTTTGGGATGCC | BamHI |
| DT66 | 5‘‑TTTGTCGACGTTTACCCCGATATATTGATCTTCAACCG | SalI |
| **Construction of GFP-fusion proteins** | |  |
| ***yloU*-GFP** | |  |
| DT23 | 5‘‑AAAGGATCCGTGTCCATTGAATTAAGAACGAAGTACGG | BamHI |
| DT30 | 5‘‑TTTCTGCAGCGGGTTCGTCACTCGTACGC | PstI |
| ***yqhY*-GFP** | |  |
| DT31 | 5‘‑AAAGGATCCACGGAAAAACGAGAGATGAAGCGATTG | BamHI |
| DT32 | 5‘‑TTTCTGCAGCATTTCTTCGTCGATTTGGACTTCTTGG | PstI |
